# Supplementary material for: Single-cell quantification and dose-response of cytosolic siRNA delivery
Source: Nat Commun. 2023 Feb 25;14:1075. doi: 10.1038/s41467-023-36752-1 (PMC9968291; doi:10.1038/s41467-023-36752-1)
Supplement: Supplementary file 2 — Reporting Summary [file 41467_2023_36752_MOESM2_ESM.pdf]

nature portfolio

Corresponding author(s): Anders Wittrup

Last updated by author(s): Feb 13, 2023

Reporting Summary

Nature Portfolio wishes to improve the reproducibility of the work that we publish. This form provides structure for consistency and transparency in reporting. For further information on Nature Portfolio policies, see our Editorial Policies and the Editorial Policy Checklist.

Statistics

For all statistical analyses, confirm that the following items are present in the figure legend, table legend, main text, or Methods section.

n/a	Confirmed	
☐	☒	The exact sample size (*n*) for each experimental group/condition, given as a discrete number and unit of measurement
☐	☒	A statement on whether measurements were taken from distinct samples or whether the same sample was measured repeatedly
☐	☒	The statistical test(s) used AND whether they are one- or two-sided<br>*Only common tests should be described solely by name; describe more complex techniques in the Methods section.*
☒	☐	A description of all covariates tested
☒	☐	A description of any assumptions or corrections, such as tests of normality and adjustment for multiple comparisons
☐	☒	A full description of the statistical parameters including central tendency (e.g. means) or other basic estimates (e.g. regression coefficient) AND variation (e.g. standard deviation) or associated estimates of uncertainty (e.g. confidence intervals)
☒	☐	For null hypothesis testing, the test statistic (e.g. *F*, *t*, *r*) with confidence intervals, effect sizes, degrees of freedom and *P* value noted<br>*Give P values as exact values whenever suitable.*
☒	☐	For Bayesian analysis, information on the choice of priors and Markov chain Monte Carlo settings
☒	☐	For hierarchical and complex designs, identification of the appropriate level for tests and full reporting of outcomes
☒	☐	Estimates of effect sizes (e.g. Cohen's *d*, Pearson's *r*), indicating how they were calculated

Our web collection on statistics for biologists contains articles on many of the points above.

Software and code

Policy information about availability of computer code

Data collection	ZEN 2.1 (black), BD Accuri C6 Software Version 1.0.264.21, StepOne Software v.2.3.
Data analysis	MATLAB R2018b, Cell Profiler 2.2.0, Cell Profiler Analyst 2.0, Microsoft Excel for Mac Version 15.30, GraphPad Prism 8 for Mac Version 8.4.3 and GraphPad Prism 9 for Mac Version 12.6, Fiji 1.53c, Adobe Illustrator version 23.0.2, Adobe Premiere Pro version 23.1.0. Custom MATLAB code was used to analyze absolute siRNA quantification and knockdown kinetics in single cells. Custom software is available at publication at github.com/WittrupLab/CytosolQuant.

For manuscripts utilizing custom algorithms or software that are central to the research but not yet described in published literature, software must be made available to editors and reviewers. We strongly encourage code deposition in a community repository (e.g. GitHub). See the Nature Portfolio guidelines for submitting code & software for further information.

Data

Policy information about availability of data

All manuscripts must include a data availability statement. This statement should provide the following information, where applicable:

- Accession codes, unique identifiers, or web links for publicly available datasets
- A description of any restrictions on data availability
- For clinical datasets or third party data, please ensure that the statement adheres to our policy

Source data of all quantitative figures are provided in the Source data file. All data supporting the findings of this study are available at publication at github.com/WittrupLab/CytosolQuant or from the corresponding author upon reasonable request.

Human research participants

Policy information about studies involving human research participants and Sex and Gender in Research.

Reporting on sex and gender	No studies on human research participants were performed in this manuscript.
Population characteristics	*Describe the covariate-relevant population characteristics of the human research participants (e.g. age, genotypic information, past and current diagnosis and treatment categories). If you filled out the behavioural & social sciences study design questions and have nothing to add here, write "See above."*
Recruitment	*Describe how participants were recruited. Outline any potential self-selection bias or other biases that may be present and how these are likely to impact results.*
Ethics oversight	*Identify the organization(s) that approved the study protocol.*

Note that full information on the approval of the study protocol must also be provided in the manuscript.

Field-specific reporting

Please select the one below that is the best fit for your research. If you are not sure, read the appropriate sections before making your selection.

☒ Life sciences ☐ Behavioural & social sciences ☐ Ecological, evolutionary & environmental sciences

For a reference copy of the document with all sections, see nature.com/documents/nr-reporting-summary-flat.pdf

Life sciences study design

All studies must disclose on these points even when the disclosure is negative.

Sample size	No pre-specified sample size determination was performed. Data was collected for as many data points (cells) as were technically possible.
Data exclusions	No general data exclusions were made. In the analysis pipeline exclusions of non-measurable cells were made according to pre-determined criteria as specified in the code; Cells with no or low d1-eGFP expression were excluded using a fixed value threshold Measurements in apoptotic cells were masked after apoptosis. Cells entering the field of view after the first 3 frames were excluded to prevent cells that might have had a prior undetected event to be included and wrongfully interpreted.
Replication	No formalized independent replication beyond the data presented in the manuscript was performed. Data presented are multiple collated experiments with consistent in-between experiment findings.
Randomization	Samples were not randomized. Not practical and not of value given downstream data extraction was algorithm-based, i.e., not influenceable by investigator.
Blinding	For manual release event detection (Fig 3b-3d), the individual doing the manual classification was blinded to the independent measures of release event detection. For manual inspection of de novo galectin-9 recruitment (S Fig. 6), the individual doing the inspection was blinded to the presence/absence of illumination.

Reporting for specific materials, systems and methods

We require information from authors about some types of materials, experimental systems and methods used in many studies. Here, indicate whether each material, system or method listed is relevant to your study. If you are not sure if a list item applies to your research, read the appropriate section before selecting a response.

Materials & experimental systems

n/a	Involved in the study
☒	☐ Antibodies
☐	☒ Eukaryotic cell lines
☒	☐ Palaeontology and archaeology
☒	☐ Animals and other organisms
☒	☐ Clinical data
☒	☐ Dual use research of concern

Methods

n/a	Involved in the study
☒	☐ ChIP-seq
☐	☒ Flow cytometry
☒	☐ MRI-based neuroimaging

Eukaryotic cell lines

Policy information about cell lines and Sex and Gender in Research

Cell line source(s)	HeLa from ATCC
Authentication	Genotyping according to ANSI/ATCC standard ASN-0002 (Eurofins) with STR. All used cell lines and clones have been validated.
Mycoplasma contamination	Regular mycoplasma contamination screening was performed and confirmed to be negative.
Commonly misidentified lines (See ICLAC register)	None

Flow Cytometry

Plots

Confirm that:

☒ The axis labels state the marker and fluorochrome used (e.g. CD4-FITC).

☒ The axis scales are clearly visible. Include numbers along axes only for bottom left plot of group (a 'group' is an analysis of identical markers).

☒ All plots are contour plots with outliers or pseudocolor plots.

☒ A numerical value for number of cells or percentage (with statistics) is provided.

Methodology

Sample preparation	HeLa cells
Instrument	Becton Dickinson Accuri C6
Software	BD Accuri C6 Software Version 1.0.264.21
Cell population abundance	N/A. Single population in homogenous cell line.
Gating strategy	FSC and SSC gating of main population according to supplied figure.

☒ Tick this box to confirm that a figure exemplifying the gating strategy is provided in the Supplementary Information.
