# Supplementary material for: Single-cell quantification and dose-response of cytosolic siRNA delivery
Source: Nat Commun. 2023 Feb 25;14:1075. doi: 10.1038/s41467-023-36752-1 (PMC9968291; doi:10.1038/s41467-023-36752-1)
Supplement: Supplementary file 3 — Description of Additional Supplementary Files [file 41467_2023_36752_MOESM3_ESM.pdf]

Title: Supplementary Movie 1.
Description: Cytosolic delivery of siRNA during sub-nanomolar siRNA transfection. An Airyscan confocal detector was used to image HeLa cells stably expressing d1-eGFP every 5 min during treatment with 0.4 nM lipoplexed AF647-siRNA. Scale bar is 100 µm.

Title: Supplementary Movie 2.
Description: Endosomal escape of siRNA and gradual cytosolic diffusion.
HeLa cells stably expressing YFP-galectin-9 were treated with lipoplexed AF647-siRNA and live-cell imaged every 5 s with an Airyscan confocal detector. In order of appearance, marked regions indicate YFP-galectin-9 recruitment to lipoplex-containing vesicle and AF647-siRNA redistribution into cytoplasmic foci. Scale bar is 20 µm.

Title: Supplementary Movie 3.
Description: Galectin-9 recruitment and subsequent endosomal release of siRNA into the cytosol. HeLa cells stably expressing YFP-galectin-9 were treated with lipoplexed AF647-siRNA and live-cell imaged with an Airyscan confocal detector every 5 min, followed by single cell quantification. Cells and associated AF647-siRNA fluorescent measurements are identified by color, arrow indicates de novo YFP-galectin-9 recruitment on lipoplex-containing vesicles and outline indicates cell contours at endosomal release and cytosolic dispersion. Mobile line in graph indicates current time in movie-sequence. Scale bar is 50 µm.

Title: Supplementary Movie 4.
Description: Dose-dependent single-cell knockdown kinetics.
HeLa cells stably expressing d1-eGFP were treated with 40–2000 pM lipoplexed AF647-siRNA targeting eGFP. A confocal microscope with Airyscan detector was used for live-cell imaging, followed by single-cell analysis. Normalized single-cell knockdown kinetics between 0 h and 10 h after siRNA release. Model-estimated cytosolic siRNA concentration and eGFP expression relative to $t = 0$ is shown per cell, for siGFP-1 (left) and siGFP-2 (right). Release events with model R^2>0.3 are shown.
